# Plasma Levels of MicroRNA-499 Provide an Early Indication of Perioperative Myocardial Infarction in Coronary Artery Bypass Graft Patients

**DOI:** 10.1371/journal.pone.0104618

**Published:** 2014-08-11

**Authors:** Youxiu Yao, Juan Du, Xiaoqing Cao, Yang Wang, Yaohua Huang, Shengshou Hu, Zhe Zheng

**Affiliations:** 1 Chinese Academy of Medical Sciences and Peking Union Medical College, State Key Laboratory of Cardiovascular Diseases, National Center for Cardiovascular Diseases, Peking, China; 2 Department of Cardiac Surgery, Fuwai Hospital and Cardiovascular Institute, Peking, China; 3 Key Laboratory of Cardiac Regenerative Medicine, Ministry of Health, National Center for Cardiovascular Diseases, Peking, China; 4 Department of Thoracic Surgery, Beijing Tuberculosis and Thoracic Tumor Research Institute, Beijing Chest Hospital, Capital Medical University, Tongzhou, Beijing, China; Tokai University, Japan

## Abstract

**Background:**

Recent studies indicated that microRNAs (miRNAs, miRs) were important for many biological and pathological processes, and they might be potential biomarkers for cardiovascular diseases. The present study aims to determine the release patterns of miRNAs in cardiac surgery and to analyze the ability of miRs to provide early prediction of perioperative myocardial infarction (PMI) in patients undergoing coronary artery bypass graft (CABG) surgery.

**Methodology/Principal Findings:**

Thirty on-pump CABG patients were recruited in this study; and miR-499, miR-133a and miR-133b, cardiac troponin I (cTnI) were selected for measurement. Serial plasma samples were collected at seven perioperative time points (preoperatively, and 1, 3, 6, 12, 24, and 48 hours after declamping) and were tested for cTnI and miRs levels. Importantly, miR levels peaked as early as 1–3 hours, whereas cTnI levels peaked at 6 hours after declamping. Peak plasma concentrations of miRs correlated significantly with cTnI (miR-499, r = 0.583, P = 0.001; miR-133a, r = 0.514, P = 0.006; miR-133b, r = 0.437, P = 0.05), indicating the degree of myocardial damage. In addition, 30 off-pump CABG patients were recruited; miR-499 and miR-133a levels were tested, which were significantly lower in off-pump group than in on-pump group. A prospective cohort of CABG patients (n = 120) was recruited to study the predictive power of miRs for PMI. The diagnosis of PMI strictly adhered to the principles of universal definition of myocardial infarction. The data analysis revealed that miR-499 had higher sensitivity and specificity than cTnI, and indicated that miR-499 could be an independent risk factor for PMI.

**Conclusion:**

Our results demonstrate that circulating miR-499 is a novel, early biomarker for identifying perioperative myocardial infarction in cardiac surgery.

## Introduction

Perioperative myocardial infarction (PMI), as the most common cause of serious complications, occurs in 3% to 21% of patients undergoing surgery for coronary heart disease, leads to great postoperative morbidity and mortality and results in a considerable impact on the length and cost of hospitalization [1], [2]. As the population ages, increasing numbers of high-risk cardiovascular patients will undergo surgery and sustain PMI’s [3]. However, at present, as there are no obvious symptoms in sedated patients, it is difficult to diagnose PMI in the early postoperative period. In addition, ECG signs may also be unreliable because abnormal findings may be absent or may be non-specific [4]. Traditionally, PMI is often recognized late (postoperative day 3 to 5), resulting in high (30% to 70%) mortality [3]. Currently, although monitoring of the increase of cardiac troponin I (cTnI) level has helped to improve the diagnosis of PMI at 24 to 48 hours of surgery [1], an earlier diagnosis that potentially further reduces the mortality rate is still desirable.

MicroRNAs (miRNAs) are a class of conserved, single-stranded non-coding RNAs consisting of 19–25 nucleotides that regulate a variety of biological cell behaviors of proliferation, differentiation, and development [5]–[7]. It seems clear that some miRNAs are present in a tissue- or cell-specific manner, although the biological functions of miRNAs are only partly understood [7]. Recent studies have demonstrated that miRNAs are also present in various body fluids, including plasma or serum, and circulating miRNA levels have emerged as novel biomarkers linked to various diseases [7], [8]. Recent reports suggest that the plasma levels of heart-expressed miRNAs respond to cardiac injury similarly to cardiac enzymes, and several groups have reported circulating miRNA levels are increased in patients with acute myocardial infarctions [9]–[12], indicating that plasma miRNAs are potentially new and sensitive biomarkers.

With the emerging role of circulating miRNAs in the diagnosis of various diseases [13]–[15] and their early appearance in circulation [9]–[12], the objective of the present work was to examine the release patterns of heart-specific miRNAs and analyse whether miRNAs can provide early prediction of PMIs after cardiac surgery.

A two-step study was conducted on CABG patients. In step I, we selected cardiac-specific miRs as candidates for the present study, tested their release patterns and relevance to myocardial injury, and compared miRs release between on-pump group and off-pump group. In step II, PMI was diagnosed according to the statements of Joint Task Force of ESC/ACCF/AHA/WHF, the PMI diagnostic ability of miRs was tested and we performed a multivariate analysis.

## Methods

### Ethics statement

The study has been approved by the Ethical Committee of FuWai Hospital, Chinese Academy of Medical Sciences, and adhered to the tenets of the Declaration of Helsinki. In addition, all patients provided informed written consent prior to cardiac surgery was carried out.

### Participants and study design

**Figure 1:**
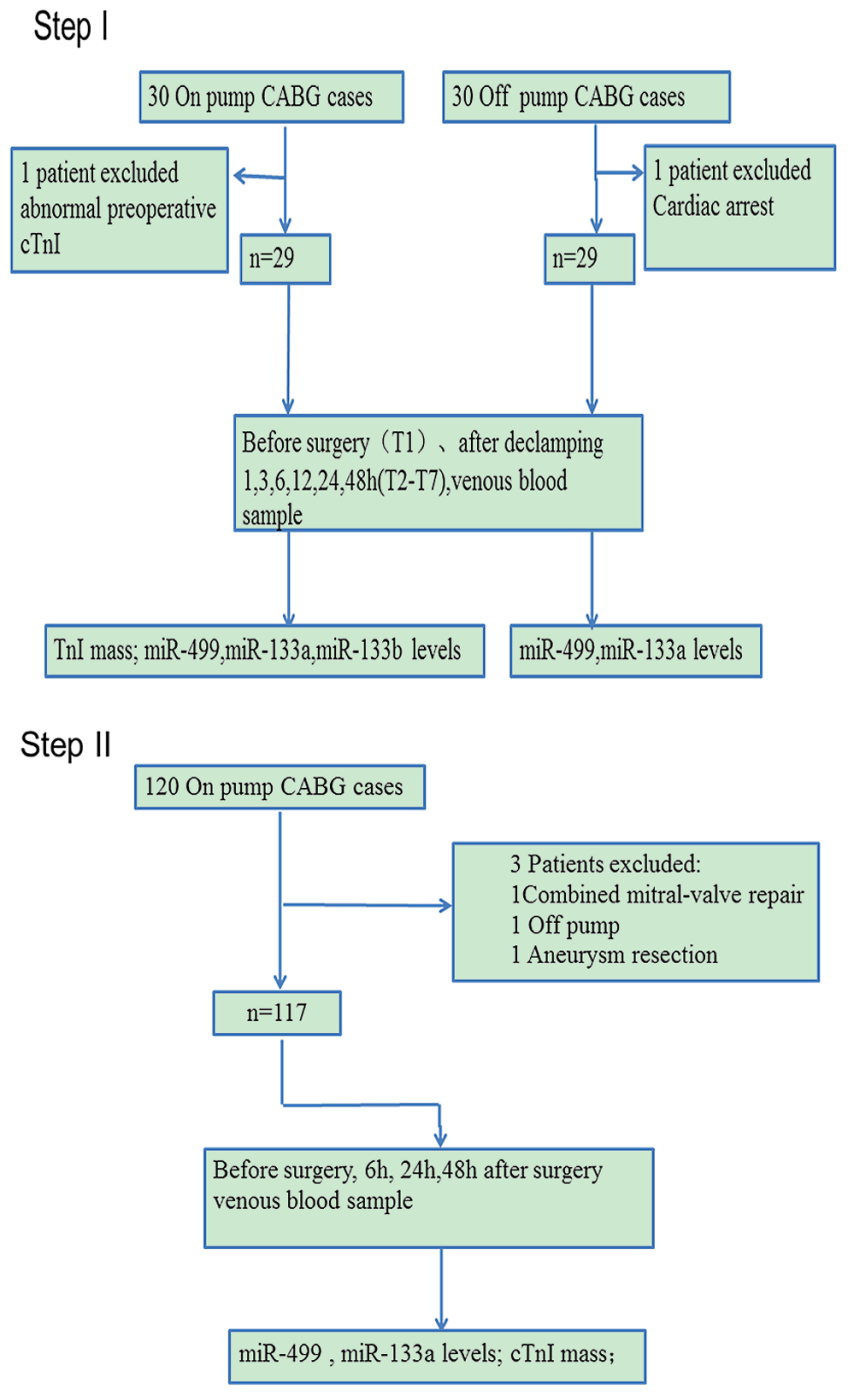
Study protocol. CABG, coronary artery bypass graft.

The study population included adult patients, referred for coronary artery bypass graft surgery at Fu Wai Hospital between October 2012 and July 2013. Emergencies, reoperations, abnormal preoperative cTnI and combined procedures were excluded. The clinical data of the selected patients are outlined in detail in Table 1 and Table 2. The study design is shown in Figure 1. In step I, 30 consecutive patients scheduled for on-pump CABG were enrolled. MiR-133a, miR-133b and miR-499 levels were serially detected from plasma samples. We studied their release patterns and their association with myocardial damage. In addition, 30 off-pump CABG patients were recruited. We tested and compared the miR-499 and miR-133a levels between the two groups.

**Table 1:** Perioperative patient characteristics and clinical data.

Parameter	CABG (n = 29)	OPCAB (n = 29)	p Value
Age (yr)	61.7±9.1	58.9±9.8	0.240
Male/female	22/7	24/5	0.534
Hypertension, n (%)	20 (69%)	18 (62%)	0.795
Diabetes, n (%)	10 (34%)	9 (31%)	0.849
Hyperlipidemia, n (%)	22 (76%)	20 (69%)	0.582
BMI (kg/m^2^)	25.9±3.0	24.9±3.2	0.195
Ejection fraction (%)	60.2±9.0	62.6±7.9	0.258
Smoking, n (%)	15 (52%)	18 (62%)	0.208
Alcohol, n (%)	5 (17%)	2 (7%)	0.246
CPB time (min)	113.6±30.1	––	––
Aortic clamping time (min)	75.4±20.3	––	––
Distal coronary anastomoses (no./patient)	3.51±0.91	3.17±0.76	0.123

BMI, body mass index; CPB, cardiopulmonary bypass; CABG, coronary artery bypass graft surgery; OPCAB, off-pump coronary artery bypass. The data are presented as the means (±SD) or number (%).

**Table 2:** Demographic and Perioperative Characteristics of Enrolled Patients.

Parameter	All Patients(n = 117)	Patients withPMI (n = 28)	Patients withoutPMI (n = 89)	pValue(Univariate)	pValue*
Age (yr)	60.7±9.9	61.1±10.9	60.5±10.6	0.806	
Male, n (%)	98 (84)	23 (82)	75 (86)	0.941	
Height (cm)	167.7±7.8	165.6±8.1	168.3±7.6	0.105	NS
Weight (kg)	73.1±10.6	71.3±11.7	73.6±10.2	0.305	
Preoperative LVEF (%)	58.7±9.7	54.3±11.2	60.1±8.9	0.005	NS
Hypertension, n (%)	77 (66)	19 (68)	58 (65)	0.822	
Diabetes, n (%)	37 (32)	7 (25)	30 (34)	0.493	
Hyperlipidemia, n (%)	60 (51)	12 (43)	48 (54)	0.518	
Smoking, n (%)	60 (51)	15 (54)	45 (51)	0.829	
Prior myocardial infarction, n (%)	39 (33)	13 (46)	26 (29)	0.106	NS
CCS	2 (2–3)	2.5 (2–3)	2 (2–3)	0.429	
NYHA	2 (1–3)	2 (2–3)	2 (2–3)	0.839	
ACE inhibitor, n (%)	59 (50)	15 (53)	45 (51)	0.756	
β Blocker, n (%)	80 (68)	26 (75)	54 (61)	0.445	
Distal coronary Anastomoses (no./patient)	3 (3–4)	3 (3–4)	3 (3–4)	0.877	
Cardiopulmonary bypass (min)	101.1±31.5	114.6±39.3	96.0±26.7	0.009	NS
Aortic cross clamp (min)	69.3±22.7	78.2±24.8	65.9±21.0	0.017	NS
Postoperative Mechanical Ventilation (h)	17.5(15–23)	22.5(17–36.5)	17(15–20)	0.000	NS
Troponin I (ng/ml)	1.84(1.22–3.14)	4.03(2.23–6.56)	1.64(1.11–2.53)	0.000	NS
MiR-499 Δct	19.79(18.74–21.03)	17.18(16.20–18.20)	20.14(19.50–21.79)	0.000	0.002
MiR-133a Δct	13.79(12.94–14.86)	12.67(11.00–13.44)	14.14(13.40–14.95)	0.000	NS

LVEF, left ventricular ejection fraction; CCS, Canadian classification score for angina grade; NYHA, New York Heart Association grade for heart failure. ACE, angiotensin-converting enzyme.

*multiple logistic regression analysis (only variables with p<0.2 by univariate analysis included).

NS = not significant.

In step II, a prospective cohort of 120 on-pump CABG patients was recruited. We performed diagnostic tests with a multivariate analysis on this cohort. PMIs were diagnosed on the basis of the following signs according to the diagnostic criteria for myocardial infarction with CABG (MI type 5) by the Joint ESC/ACCF/AHA/WHF Task Force for the Third Universal Definition of Myocardial Infarction [16]: Myocardial infarction associated with CABG is arbitrarily defined by elevation of cardiac biomarker values (>10×99th percentile URL) in patients with normal baseline cTn values (≤99th percentile URL). In addition, either (i) new pathological Q waves or new LBBB or (ii) angiographically documented new graft or new native coronary artery occlusion or (iii) imaging evidence of new loss of viable myocardium or new regional wall motion abnormality.

### Isolation of human plasma

Serial central venous blood samples were collected immediately after anesthesia induction and 1 h, 3 h, 6 h, 12 h, 24 h, and 48 h after declamping for biochemical analysis as indicated below. Samples were drawn into vacuum tubes containing EDTA and were immediately iced. The plasma was separated by centrifugation at 1500×g at 4°C for 10 minutes, promptly transferred to a fresh RNase/DNase-free Eppendorf tube and re-centrifuged at 14,000×g at 4°C for 15 minutes to pellet the remaining cellular debris. The supernatant was carefully transferred to fresh RNase/DNase-free tubes for extraction of RNA and stored at –80°C until further use.

### RNA extraction and real-time quantitative RT-PCR (qRT-PCR)

Total RNA was isolated from 400 µl of plasma using a miRVana PARIS isolation kit (ABI) following the manufacturer’s instructions for liquid samples with modification: 0.376 µl of synthetic *C. elegans* miRNA (cel-miR-39, 10 ng) was spiked-in as a control before chloroform extraction, as described previously [17]. Lastly, the total RNA was eluted in 50 µl of nuclease-free water [18].

RNA (4.5 µl) was used per 10 µl reaction to generate cDNA with the TaqMan MicroRNA Reverse Transcription Kit (Applied Biosystems, Cat. #4366597) and miRNA-specific stem-loop primers following the manufacturer’s instructions (Applied Biosystems). The primers were designed to specifically detect mature microRNAs. cDNA(2 µl) was added per quantitative reverse transcriptase-polymerase chain reaction. A TaqMan Universal PCR Master Mix II (Applied Biosystems, Cat. #4440040) was used for relative quantification of miRNAs in our study with synthesized cel-39 as the spiked-in control [19]. qRT-PCR was performed with a 7900 FAST Real-Time PCR System (Applied Biosystems). The threshold cycle (Ct) is defined as the fractional cycle number at which the fluorescence exceeds the given threshold. The Ct values from PCR assays greater than 40 were treated as 40. The results were expressed as Cts and normalized to the calculated median Ct of each sample (ΔCt). The relative expression was calculated using the comparative Ct method (2^−ΔΔCt^).

### Cardiac troponin I mass determination

MiR and cTnI levels were measured in the same plasma samples. Plasma levels of cTnI were determined by electrochemiluminescence-based methods and a Beckman ACCESS2 Analyzer. The upper limit for the normal reference range was 0.04 ng/ml.

### Statistical analysis

Continuous variables were expressed as the mean ± standard deviation or median (Interquartile range, 25% to 75%) as appropriate. Categorical variables were described by counts and percentages. Plasma miRNA levels were presented as the fold-change relative to the controls. Linear regression models were used to clarify the relationships between miRs and the diagnostic components of the baseline or cTnI levels. The mean miRNA levels of CABG patients were compared at seven time points with the baseline using repeated measures ANOVA. Bonferroni correction was performed for multiple testing when continuous variables were compared between groups. Receiver operating characteristic (ROC) curves were established for diagnosing patients with PMI, and a multivariate logistic regression analysis was performed. An alpha of 0.05 was set as the level of statistical significance. All analyses were performed with GraphPad Prism 5.0 and MedCalc statistical software.

## Results

### The mean concentration of miRs in CABG patients

**Figure 2:**
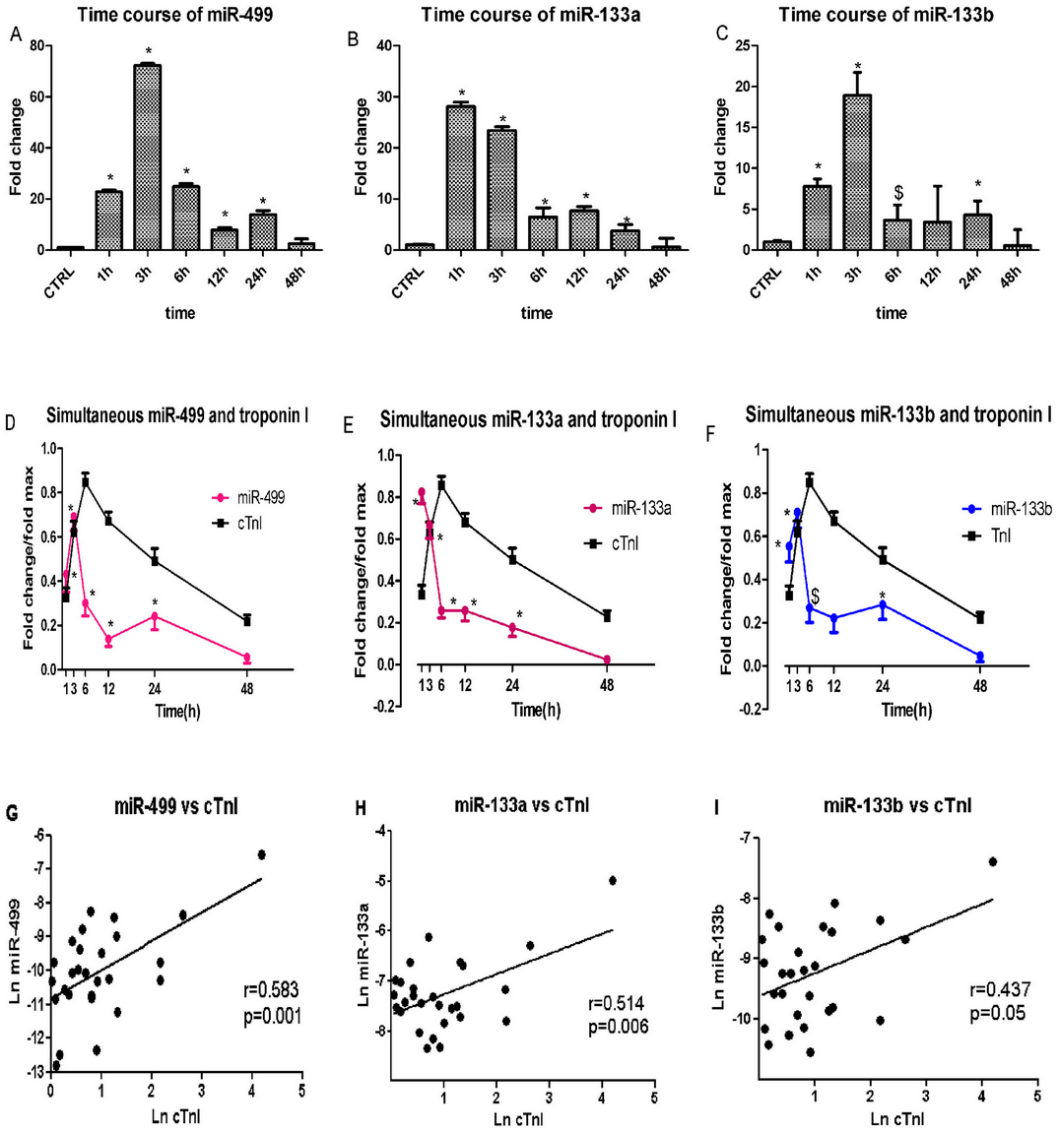
Plasma miR and troponin I levels from 29 patients undergoing on-pump CABG (Step I). (A–C) Mean concentrations of circulating miRs expressed as the fold increase (2^−ΔΔCt^ scale) relative to those at the preoperative time point. On average, miR-499, miR-133a, and miR-133b exhibited a 20- to 70-fold increase in plasma samples collected 1 h and 3 h after declamping, respectively. At 48 h after declamping, the values were back to levels comparable to those in the preoperative control. (D–F) Comparison of miRs and cTnI levels in the same plasma samples showing that miR-499, miR-133a and miR-133b peaked earlier than cTnI. (*P<0.01; ^$^P<0.05 vs. control) (G–I) Correlations between the maximum levels of miRs and cTnI. MiR-499 (G), miR-133a (H) and miR-133b (I) correlated significantly with cTnI levels (respectively P<0.001; P<0.01 and P<0.05).

The circulating levels of miR-499, as indicated by the fold-change values, were highly elevated at 1, 3, and 6 h after declamping in CABG patients. The levels were 23-fold (P<0.01), 72-fold (P<0.01), and 25-fold (P<0.01) higher, respectively, than the preoperative control level (Fig. 2A). Strikingly, the increased circulating level of miR-499 was restored back to the control value by 48 h after declamping (2-fold, P>0.05, Fig. 2A). MiR-133a and miR-133b levels also increased, though to a lesser extent (Fig. 2B, C).

### Time course of simultaneous miRs and cardiac troponin I plasma levels

Both miRs and cTnI displayed typical release patterns. The concentrations of both rose from the baseline value to postoperative peak in all patients. Thereafter, the concentrations declined and reached baseline values by 48 h. The miRs values increased rapidly and reached a peak at 1–3 h after declamping. The elevated level of miRs displayed a sharp decline at 6 h and a biphasic pattern with a second peak at 12–24 h, before returning to basal levels 48 hrs after declamping. Importantly, the miRs achieved their peak at 1–3 h, whereas cTnI peaked later at 6 h (Fig. 2D, E, F). Thus, miRs have an earlier time course than the traditional protein biomarker troponin I.

### Correlation between maximum level of miRs and extent of myocardial Injury

To further document an association between circulating concentrations of miRs and myocardial injury, we correlated cTnI levels with individual miRs levels. As illustrated in Figure 2G, H, I, a significant correlation was observed between the peak values of miRs and cTnI (miR-499, r = 0.583, P = 0.001; miR-133a, r = 0.514, P = 0.006; miR-133b, r = 0.437, P = 0.05). Thus, the release of the cardiac-enriched miR-499 and miR-133a reflected the extent of myocardial injury as measured by troponin release into circulation. An analysis with univariate linear regression models ruled out a correlation between age/gender/BMI and circulating miR-499 or miR-133a concentration (P>0.05, data not shown). Linear regression analyses revealed that plasma miRs levels were not significantly associated with extracorpereal circulation time, clamping time, or number of bypass grafts (P>0.05, data not shown). Other possible clinical variables, such as diabetes mellitus, hypertension, and hyperlipemia were also insignificantly correlated. Only the level of preoperative ejection fraction significantly correlated with miR-499 or miR-133a levels (P = 0.022, data not shown). We selected miR-499 and miR-133a for our next study.

### On-pump vs. off-pump

**Figure 3:**
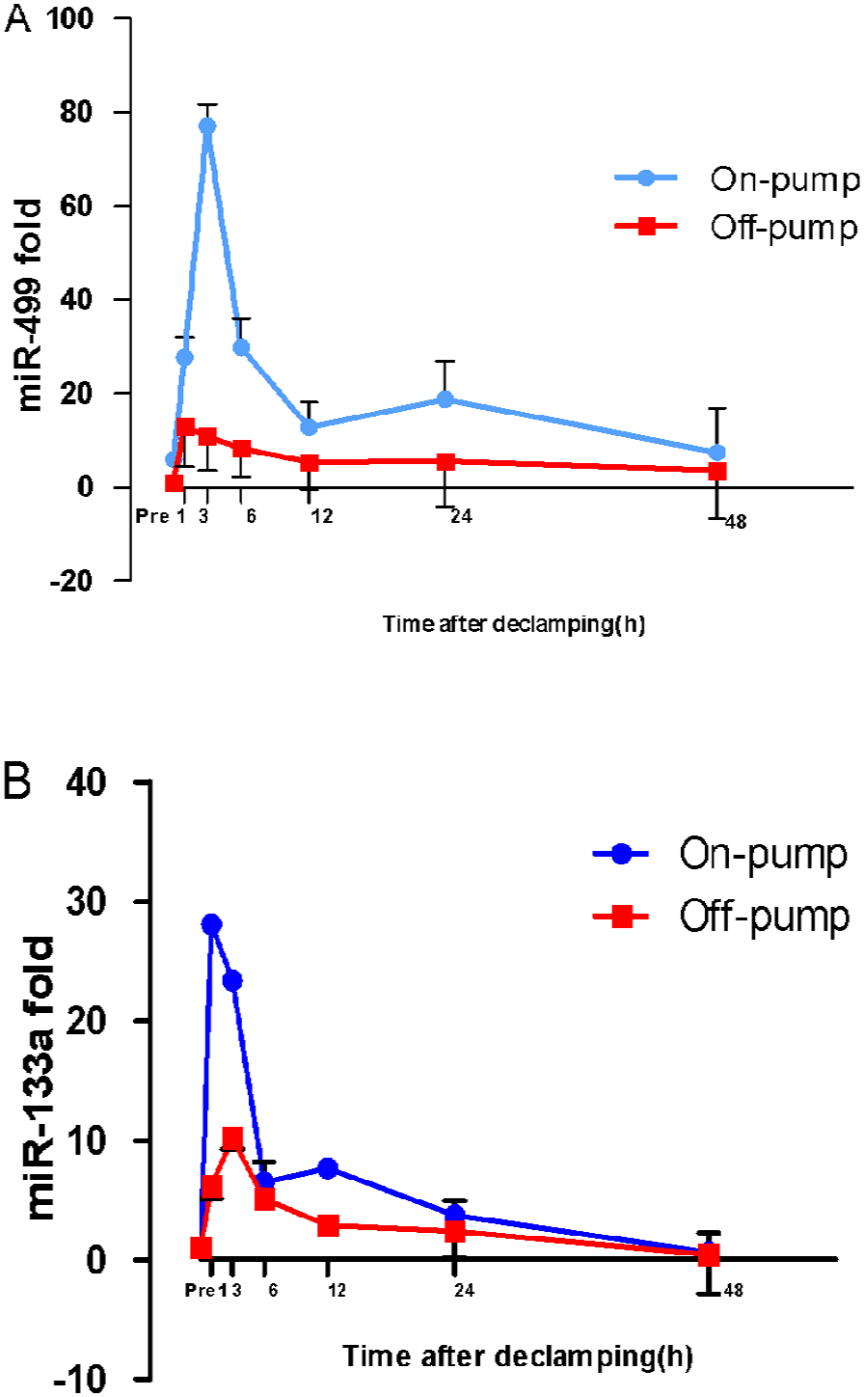
Pump-on and pump-off differences in miR-499 (**Figure 3A**) and miR-133a (**Figure 3B**) concentrations. The two curves differ significantly (p<0.0001) for both miR-499 and mIR-133a. In the arrested heart (on-pump) group versus the beating heart (off-pump) group, the miR-499 concentrations were significantly higher at post-declamping hours 1, 3, 6, 12, and 24 (p<0.0001), while the miR-133a concentrations were significantly higher at hours 1, 3, and 12 (p<0.0001). All mean±standard deviation levels are expressed as fold increase (2^−ΔΔCt^ scale) relative to those in the preoperative control.

To differentiate the release of miRs in myocardial ischemia reperfusion injury from the surgical thoracotomy procedure and cardiac manipulation, we conducted an additional study with the following two groups: the whole heart ischemia-reperfusion surgery (on-pump group) and surgery on the beating heart (off-pump group). Figure 3 shows the time course of miR-499 and miR-133a levels according to the surgical procedure. Overall, the two curves differ significantly (p<0.001). As expected, the levels of miR-133a and miR-499 were higher in plasma from the on-pump group compared with plasma from the off-pump group in the first 48 hours after CABG. For each sample drawn, miR-499 concentrations were significantly higher (p<0.001) in the on-pump group than in the off-pump group at 1 h, 3 h, 6 h, and 24 h: 22.76±4.19-fold vs 13.02±8.66-fold at 1 h, 72.14±4.62-fold vs 10.89±7.26-fold at 3 h; 24.82±6.26-fold vs 8.39±6.16-fold at 6 h, and 13.83±8.17-fold vs 5.63±9.76-fold at 24 h (Fig. 3A). The miR-133a level was also significantly higher (p<0.001) in the on-pump group, although the difference between the two groups were not as obvious as with miR-499: 28.08±4.68-fold vs 6.21±5.73-fold at 1 h, 23.40±3.79-fold vs 10.20±5.00-fold at 3 h, and 7.69±4.50-fold vs 2.88±1.22-fold at 12 h (Fig. 3B).

### Proposed predictive levels of circulating miRs for PMI

The demographic data and perioperative data of the patients in this group are presented in Table 2. Twenty-eight patients had PMI according to the diagnostic criteria of the Joint ESC/ACCF/AHA/WHF Task Force for the Third Universal Definition of Myocardial Infarction [16]: 19 had new Q waves, 2 had new LBBB, 10 had new regional wall motion abnormality, and none had angiographic documented new graft or new native coronary artery occlusion. The independent predictive value of the plasma markers and other important clinical variables are also presented in Table 2. Univariate analysis revealed that cTnI, miR-499, miR-133a, preoperative LVEF, cardiopulmonary bypass time, aortic cross clamping time and postoperative mechanical ventilation time were significantly associated with PMI. In a multivariate statistical model, only miR-499 was found to be significantly independently diagnostically associated (p = 0.002).

**Figure 4:**
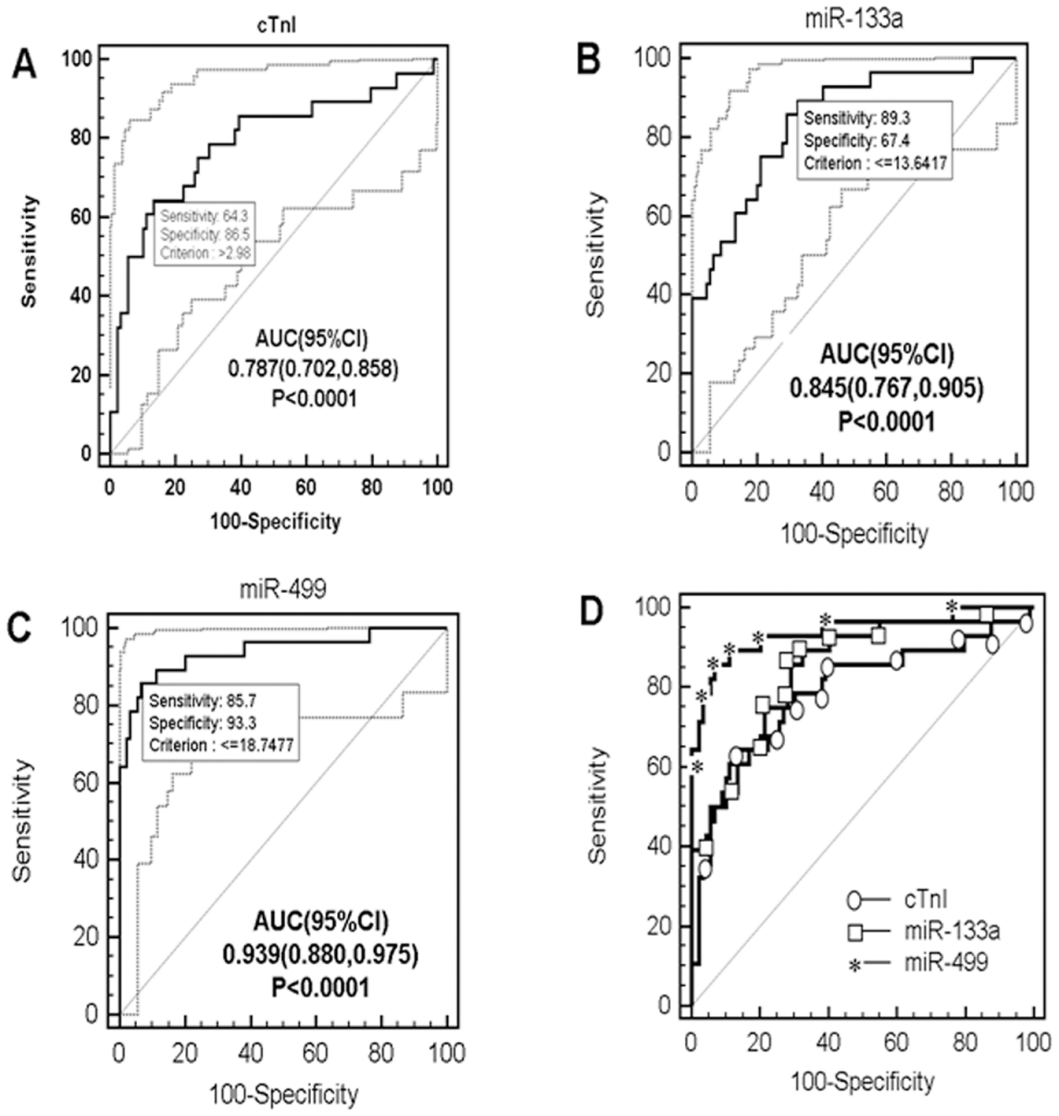
Receiver operating characteristic(ROC) curves for the identification of PMI at 6 hours after CABG. The thin diagonal line is the Null Hypothesis: True area = 0.50. A, B and C panels show the ROC and AUC values of cTnI, miR-133a and miR-499, respectively, as thick solid lines. The dotted lines show the 95% CI of ROC curves. The highest AUC was calculated from miR-499 (0.939, p<0.0001). (A) cTnI (>2.98 ng/ml) had a sensitivity of 64.3% and specificity of 86.5% for the **identification** of PMI. (B) miR-133a (ΔCt<13.6417) had a sensitivity of 89.3% and specificity of 67.4% for the **identification** of PMI. (C) miR-499 (ΔCt<18.7477) had a sensitivity of 85.7% and specificity of 93.3% for the **identification** of PMI. (D) Comparison of ROC curves of cTnI(*ellipses*), miR-133a(*rectangles*) and miR-499 (*asterisks*) at 6 h postoperatively. Abbreviations: AUC = area under the curve; Ct = cycle threshold; cTnI = cardiac troponin I; PMI = perioperative myocardial infarction.

ROC curve analysis indicated that miR-499 accurately diagnosed PMI, with a sensitivity of 85.7%, specificity of 93.3% and area under the curve (with 95% confidence interval) of 0.939 (0.880–0.975). A threshold ΔCt value of <18.7477 at 6 h defined a group at risk for PMI. The miR-133a value was 89.3% sensitive and 67.4% specific, and the area under the ROC curve (with 95% confidence interval) was 0.845 (0.767–0.905). As for cTnI, for a cutoff value of 2.98 ng/ml, the sensitivity was only 64.3% and the specificity 86.5%, with an area under the ROC curve of 0.787 (0.702–0.858) (Fig. 4).

The correlations between miRs and cTnI in this group of patients follow. Plasma levels of miR-499 and miR-133a were positively correlated with cTnI concentrations at the same timepoint (r = 0.379, p = 0.000 and r = 0.315, p = 0.001, respectively, data not shown). Figure 4 and Table 2 also show the distribution of cTnI and miRs ΔCt values in the two groups of patients (with or without PMI) at 6 h after surgery with the selected cutoffs from the ROC curves. Using the cutoffs defined by the ROC approach, we calculated the likelihood ratios for PMI and their 95% confidence intervals. The likelihood ratios for miR-499, miR-133a and cTnI were 12.71(10.8–14.9), 2.74(2.3–3.3) and 4.77(3.6–6.4), respectively, for this cohort of patients with a positive test result.

## Discussion

The present study provides the first insights into circulating miRs in cardiac surgery. We demonstrated the time course of circulating miRs in cardiac injury and clarified their correlation to the traditional marker cTnI. We demonstrated that circulating miRs, consistent with the results of a previous study of acute myocardial inarction patients, appeared earlier than the protein marker cTnI in the blood, peaked and quickly declined following on-pump CABG surgery.

As both on-pump and off-pump techniques were equally used for CABG, we further detected the time course of miRs in off-pump CABG group and compared them between the two groups. We found that on-pump group can cause a high level of miR release, far higher than observed in the beating heart (off-pump) group. Our results indicate that the level of miR-499 at 6 hours after surgery can clearly predict the occurrence of PMI, with a very high sensitivity and specificity. These results reveal, for the first time, that miR-499 is potentially a stronger biomarker than troponin in predicting PMI in the early postoperative period.

### Circulating miRs elevation during cardiac surgery

Myocardial ischemia during cardiac surgery results in functional and structural changes and ultimately the release of intracellular components into the blood. Myoglobin, creatinine kinase (especially creatinine kinase-MB isoenzyme, CKMB), and fibrillar proteins such as troponin I or T are commonly measured. However, the time delay between the detection of circulating protein and ischemic damage remains a major problem [20]. Most of the currently available biomarkers for clinical applications are proteins and polypeptides [21]. Current advances in molecular techniques have facilitated the discovery of new biomarkers such as miRs for the evaluation of myocardial ischemia-reperfusion injury during cardiac surgery.

In the present study, real-time PCR analysis revealed that the levels of miR-133a, miR-133b and miR-499 in the plasma of CABG patients rapidly increased and peaked within 1–3 h and decreased at 6 h after aortic declamping. Importantly, peak increases in miRs, especially miR-499 and miR-133a, correlated significantly with peak levels in cTnI. The increased levels of circulating miRs after CABG reflect and parallel the extent of myocardial damage as measured by cardiac troponin I.

MiRNAs are bound to protein complexes that are predominantly soluble in the cytoplasm [22], which may affect their release patterns when cell injury occurs. The present study confirms and extends previous reports demonstrating that circulating miRNAs can be reliably measured in humans [17], [23], [24] and that plasma concentrations of miRs significantly increase in patients with acute myocardial infarction [18], [25]. Importantly, our results demonstrated that miRs including, miR-499, miR-133a, and miR-133b, were more rapidly washed out and detectable in the blood than cTnI during the early stage of myocardial injury in CABG (see above). Interestingly, D’Alessandra et al. [9] demonstrated that miR-499 exhibited a slower time course than cTnI in ST-segment elevation myocardial infarction patients. In our study, miR-499 peaked at 1 h after reperfusion in 30% of patients undergoing CABG operations, and nearly 90% of patients had peak levels by 3 h, which is indeed earlier than cTnI.

The present study cannot provide molecular insights into the cause of the dysregulation. The exact source of elevated levels of circulating miRNA remains unknown. The circulating miRNAs level may be affected by multiple factors including the altered expression in the tissue, the amount and rate of tissue release into the blood and the stability of circulating miRNAs [26]. A recent study that measured concentration gradients across coronary circulation for heart-enriched miRNAs indicated that miRs were released from the heart into coronary circulation upon myocardial injury [27]. Another report indicated that miRNAs were released from infarcted and peri-infarcted myocardium, suggesting that the release of miRNAs into the blood is dependent on the rupture of myocytes [28]. The latest report by Xiao and colleagues showed that serum miR-499 was associated with acute MI and was significantly decreased in infarcted than remote zones, and miR-499 might be released from damaged heart tissues to circulation [29].

Since miR-133a and miR-499 are also expressed in skeletal muscle, their increased levels in plasma might be resulted from skeletal muscle in addition to myocardial damage during surgery [11]. And why on-pump surgery demonstrates higher degree of miRs release than off-pump surgery? This can be explained by three reasons: first, the release of miRs from skeletal muscles because of low perfusion and inflammatory response during cardiopulmonary bypass; second, release from the heart because of cardioplegia and ischaemia-reperfusion; and third, because of hypothermia.

In this study, we took blood samples at seven different time points during perioperative period applicable in a normal clinical routine without conflict. Usually, the operation approaches its end at 1 h after aortic declamping and the patient’s condition is stabilized at 3 h after aortic declamping. Since the ICU staff has time for sophisticated diagnosis and treatment, early measurements of miRs could result in earlier diagnosis and possible reductions in the complications of PMI.

### Priority of early warning PMI

During CABG, numerous factors can lead to periprocedural myocardial injury, including whole heart ischemia-reperfusion injury and cardiac manipulation. During perioperative period, large, unpredictable and nonphysiological changes may be induced by changes in coronary plaque morphology and function, which may trigger myocardial ischemia [30] leading to perioperative myocardial infarction and the release of necrosis biomarkers. The diagnosis itself is lagging, and there are relatively few current studies concerning myocardial infarction associated with CABG. Recently the Joint ESC/ACCF/AHA/WHF Task Force for the Redefinition of Myocardial Infarction in 2007 [31] and Third Universal Definition of Myocardial Infarction in 2012 [16], change the level of biomarkers for PMI diagnosis from 5 to 10×99th percentile URL during the first 48 h following CABG. In fact, our results demonstrate cTnI often reachs the 10×99th percentile URL in CABG surgery without evidence of PMI. We believe that it is valuable to identify and measure a biomarker other than troponin to diagnose PMI earlier, enabling more efficient therapeutic interventions and reducing associated morbidity and mortality.

As noted in the last paragraph of the Results section and Figure 4 ROC analysis demonstrated the superior sensitivity and specificity of miR-499 over miR-133a over cTnI in the early detection of myocardial necrosis after CABG.

## Limitations

Our data are from a relatively small sample size without long-term followup. Further larger cohort studies would be desirable to confirm the utility of miR-499 and related miRNAs as a novel class of diagnostic and prognostic biomarkers for PMI in clinical daily practice. The finding that circulating cardiac-specific miR-499 correlates with the ejection fraction and peak cTnI might possibly imply a broader role for this molecule as a biomarker for the risk of morbidity and mortality other than after cardiac surgery. Whether miRs can be clinically applied also depends on the further development of approaches for simple and rapid detection of miRNAs in blood and the re-evaluation of the wisdom of dichotomous cut-off values.

## Conclusion

Early postoperative miR-499 measurements provide an early indication of perioperative myocardial infarction in aortocoronary bypass surgery.
